# Effect of high sugar levels on miRNA expression. Studies with galactosemic mice lenses

**Published:** 2012-06-17

**Authors:** Shambhu D. Varma, Svitlana Kovtun, Kavita Hegde, Jing Yin, Jamuna Ramnath

**Affiliations:** 1 Department of Ophthalmology and Visual Sciences, University of Maryland, Baltimore, MD; 2 Department of Biochemistry and Molecular Biology, University of Maryland, Baltimore, MD; 3 Biopolymer Genomic Core Facility, University of Maryland, Baltimore, MD; 4 QIAGEN Technical Service, Valencia, CA

## Abstract

**Purpose:**

Development of cataract is associated with apoptotic death of the lens epithelial cells. The purpose of this investigation was to examine whether this could be explained by enhancement in the expression of certain pro-apoptotic microRNAs (miRs), known to induce apoptosis by hybridizing with target mRNAs, with the consequence of gene silencing. In addition, it was intended to investigate if such expression could be antagonized by reactive oxygen species (ROS) scavengers.

**Methods:**

CD-1 mice weighing about 20 g were divided into three groups and fed diets, respectively, as follows: Control diet, 25% galactose diet, and 25% galactose diet containing 1% sodium pyruvate. After eight days of such a regimen, the mice were euthanized, their lenses promptly isolated and frozen in liquid nitrogen, and RNAs isolated by extraction with standard methods and converted to cDNAs. The miR-specific cDNAs were then quantified by polymerase chain reaction (PCR) using a 96-well microRNA array cassette using an ABI 7900 HT PCR machine. The results were then analyzed using Bioscience software.

**Results:**

The lens samples were positive for all of the 84 miRs expected, on the basis of the specific sequences used for amplification. However, as would be apparent from the microarray plot for the normal and galactosemic lenses, expression of at least 24 apoptotic miRs was upregulated. Six apoptotic miRs were downregulated. In the lenses of animals where the galactose diet was fortified with sodium pyruvate, the expression of 12 miRs was completely prevented. The upregulation observed in the other 14 miRs was also significantly downregulated. A comparison of the galactose and galactose plus pyruvate group clearly indicated that pyruvate inhibits the transcription of apoptotic miRS.

**Conclusions:**

The prevention of galactose-induced enhancement in the expression of apoptotic miRs by pyruvate, a compound well known to scavenge reactive oxygen species as well as to inhibit their formation, strongly suggests that the upregulation of miRs in galactosemic animals is due to generation of reactive oxygen species. This is in conformity with our previous studies showing that pyruvate and other ROS scavengers inhibit apoptosis as well as cataract formation.

## Introduction

The persistence of high blood glucose levels, particularly of monosaccharides such as glucose, galactose, and xylose, has been shown to be physiologically toxic, most apparent in the eye by the development of cataracts—the most prevalent vision impairing and blinding disease. Although the etiology is multifactorial in nature, it is generally agreed that oxidative stress concomitant to the in situ generation of various reactive oxygen species (ROS), such as the superoxide and its derivatization to hydrogen peroxide, hydroxyl radical and singlet oxygen, is responsible [1-4]. The generation of these species can be caused by pseudo-catalytic photochemical reactions induced by the continued penetration of light into the eye, as well by physiologic conditions leading to metabolic aberrations such as the persistence of high blood glucose levels, particularly glucose and galactose. The above sugars can induce the generation of these reactive species by several mechanisms, including their direct metal catalyzed auto-oxidation [5], glycation-induced inactivation of various metabolically controlled enzymes using oxygen [6,7]. In addition, there is the diversion of the respired oxygen from its regulated use in the cytochrome oxidative chain toward many cytochrome-independent auto-oxidation of highly susceptible redox active metabolites and nutrients, such as glutathione and ascorbate, present in the lens at relatively high levels. The increased use of oxygen for such unwanted reactions under hyperglycemic conditions is clearly reflected by a persistent depression of the respiratory quotient (RQ) in diabetic individuals [8], and associated with enhancements in lipid and protein degradation. The latter is apparent by increased urinary excretion of nitrogenous compounds. The increased availability of molecular oxygen in the diabetic, and other states of metabolic depression, therefore, act synergistically with the high levels of the sugars themselves, as well as with the availability of the high levels of oxidation-sensitive substances, in producing ROS. Ascorbate and glutathione, which are present at substantial levels in the lens, are highly ROS-sensitive molecules. ROS generation is also attributable to a highly significant activation of aldose reductase under hyperglycemic conditions, as compared to the normal condition wherein it remains relatively inactive [9]. This activation results in the increased flux of NADPH (nicotinamide adenine dinucleotide phosphate reduced) toward sugar alcohol (polyol) synthesis. This has an unwanted effect of altering the ratios of the oxidized to the reduced forms of the pyridine nucleotides involved in channeling tissue redox. In addition, ROS can directly deplete important tissue metabolites such as pyruvate, limiting mitochondrial ATP synthesis and consequent sparing of oxygen. The implication of oxidative stress induced by the above biochemical mechanisms in the genesis of cataracts has been proven extensively by several studies demonstrating prevention/attenuation of cataract formation by different nutritional and metabolic antioxidants in diabetic and galactosemic animal models in vivo, as well as in humans, where cataract formation has been shown to be less prevalent in groups with a higher intake of antioxidant vitamins [10].

While ROS-induced oxidative stress can become toxic by rendering many enzymatic and non-enzymatic structural and soluble proteins, lipids and their precursors dysfunctional, these effects have so far been studied primarily with regard to cell membrane damage and damage in the cytoplasmic and mitochondrial compartments. Studies on the possible involvement of nuclear elements in the above pathology have so far been limited [11-14]. However, we speculate that such an involvement exists, on the basis of the previous EM and histochemical studies [13,14] showing chromatin disorganization, including its compaction, reactivity to terminal deoxynucleotidyl transferase mediated d-UTP end labeling (TUNEL), demonstrating the presence of endonucleolytically-produced DNA nicks [15], and the presence of oligomeric DNA fractions (laddering), demonstrating nucleosomal fragmentation and its prevention by vitamin C [16]. It has been further interesting to find that these apoptotic changes are also significantly inhibited by pyruvate, ascorbate, and caffeine, which act as metabolic agonists as well as scavengers of various reactive oxygen species [17,18].

We hypothesize that these nuclear changes, including apoptotic cell death associated with cataract formation, could be attributable to an increase in the transcription of certain microRNAs and their hybridization with the target mRNAs, inhibiting the latter’s translational activities. Additionally, such complexation renders the RNAs highly sensitive to degradation by RNases. This could further magnify the toxic effect of an enhanced miR transcription. Alternatively, the miRs could act as regulators of the mRNA action.

The pathophysiological significance of microRNAs was first shown by Lee et al. [19] using *Cenorhabditis elegans*. The gene involved was named *lin-4*, and it transcribes a hairpin double-stranded RNA molecule (pri-miRNA) in the nucleus, which serves as a precursor molecule. Subsequently, the pri-miRNA is spliced into a shorter hairpin structure by nucleases known as Drosha in vertebrates and Pasha in invertebrates. These smaller hairpin structures (pre-microRNAs) are then exported to the cytoplasm, where they are spliced further by the *Dicer* gene into smaller RNA duplexes, about 22 nucleotides. long. The strands are then separated from each other in an ATP-dependent reaction [20,21]. Either of the strands can enter a silencing risk complex (RISC) and hybridize complementarily with a target mRNA in its 3′UTR, adversely affecting its translational activity. Since the requirement of complementarity for hybridization of the miRs to the UTRs of mRNAs is not highly rigid, unlike that of siRNAs, it is highly likely that miRNAs can target several mRNA species, even at the same time, with the result of a wider gene silencing and quelling effect than ordinarily expected [22,23]. Thus, they can affect the tissue physiology multifactorially.

Such diversified binding of miRs to mRNAs has therefore been suggested to be involved in the onset of several pathophysiological processes, such as in inducing oxidative stress [24-26], angiogenesis [27], atherosclerosis [28], apoptosis [29-32], and cancer development [33-40]. It is therefore likely that some synthetic anti-miRS may become useful in the therapeutic prevention of certain diseases. Such a possibility has been suggested by recent work in cancer prevention [39,40].

Thus, we hypothesize that oxidative stress and consequent apoptosis resulting in cataract formation may involve an enhancement in the transcription of certain miRs, reaching levels beyond what might be needed for normal lens development and function [41-43]. The present finding demonstrating an enhanced transcription of several miRs that are highly apoptotic is in accordance with this hypothesis. A stress response in terms of microRNA transcription was apparent, also shown by the upregulation of a limited number of anti-apoptotic miRs such as miR-21 [36,37]. It should be pointed out, however, that the action of an miR either as an anti-apoptotic or pro-apoptotic molecule may differ from one tissue to another and under one condition compared to another, dependent upon the specific mRNA targets involved [34].

The mechanism by which miRs can become up- or downregulated remains unknown. However, we have observed that this is antagonized by simultaneous feeding with pyruvate, a compound known to offset oxidative stress by its ability to scavenge various ROS species as well as inhibit their production by facilitating mitochondrial metabolism to limit unwanted ROS generation. It is thus highly possible that the observed enhancement of the transcription of certain pro-apoptotic miRs is linked to ROS generation induced by high levels of sugar, as pointed out above. While pro-apoptotic miRs can induce damage to the lens, a simultaneous preventive attempt to overcome this, although overpowered by the induction of several highly apoptotic miRs, is also indicated by the simultaneous induction of miR-21 [27], a strongly anti-apoptotic miR. This is also indicated by the downregulation of certain pro-apoptotic miRs such as miRs-142–3p,15a, 29b, 22, 32, 34c, and 144–2p in galactosemic lenses. The overall data, however, indicate that the elevation in the transcription of apoptotic miRs is far too dominant and overwhelming, with the result that the tissue succumbs to oxidative stress unless the animals are fed pyruvate.

## Methods

### Materials

CD-1 mice were obtained from Harlan Lab Inc. (Indianapolis, IN). Reagents for RNA isolation, cDNA preparation, quantitative RT^2^ PCR amplification of the cDNAs, and the miRNA-finder array were obtained from SABiosciences Corporation (Frederick, MD). Routine chemicals were obtained from Sigma-Aldrich (St. Louis, MO).

### Methods

Mice weighing about ~20 g. were fed on Purina powdered rodent chow (controls) or the above chow mixed with galactose to 25% level (group 1), or galactose plus 1% sodium pyruvate (group 2). The dietary regimen was maintained for eight days. The blood galactose level was 4±0.5 mm in both groups of galactosemic animals. The period of galactosemia was not exceeded, to minimize the extent of lens damage and leaking of materials from the tissue. The animals were then anesthetized with ketamine/xylazine mixture (6 mg/100 g ketamine and 0.75 mg/100 g xylazine) and quickly euthanized by CO_2_ inhalation. Eyes were then rapidly enucleated and dissected to isolate intact lenses atraumatically. The isolated lenses were immediately frozen by immersion in liquid nitrogen and then immediately processed for miRNA-enriched RNA isolation using a QIAGEN RNeasy reagent kit (cat. no. 217004; QIAGEN, Valencia, CA), following its protocol. Briefly, both lenses of each animal, each weighing ~8 mg, were homogenized in a lysis buffer containing phenol and guanidine thiocyanate. The lysate was then mixed with chloroform and centrifuged. The upper aqueous phase containing RNA was then aspirated, thoroughly mixed with 1.5 volumes of ethanol, and transferred quantitatively to an RNeasy mini-column attached to a 2 ml collection tube. After allowing a few minutes for equilibration and RNA binding, the column was spun, filtering out the reagents, but retaining the RNA bound to the column. Extraneous material from the column was further removed by washing buffer. The column was then attached to a new collection tube, and RNA was finally eluted by adding RNase-free (50 µl) water to the column and centrifugation. Quantification of the RNA in the eluate was then accomplished by measuring absorption at 260/280 nm. The concentration of RNA in the eluate was determined to be 350 nanograms/microliter. The quality of the RNA was ascertained electrophoretically.

First strand cDNA synthesis was performed by mixing 1.5 µg (4.3 µl) of the RNA prep with the First Strand cDNA synthesis kit (MA-03/3311401; SA Bioscience, Frederick MD) containing the primer and the reverse transcriptase in a total volume of 10 µl. After incubating the tube for 2 h at 37 °C, the tube was heated for 5 min at 90 °C to inactivate the reverse transcriptase, chilled on ice, and the volume raised to 100 µl. This was further diluted to 2,550 µl with 1,275 µl of 2× RT^2^ SYBR Green PCR Master Mix (Cat no. PA-012-12/330522; SA Biosciences) and 1,175 µl of water. This mixture (25 µl) was then added to each of the wells in the 96-well mi-Finder cassette (MAM 001; obtained from SA Bioscience). PCR amplification was performed in an ABI 7900 HT Real Time PCR machine (Applied Biosystem, Carlsbad CA) using the three-step cycling program. The resulting threshold cycle (Ct.) data for all wells was then transferred to an Excel (Microsoft, Redmond, WA) spreadsheet and exported to SABosciences for further analysis.

## Results

Apoptotic death of lens epithelial cells is known to be associated with most cataracts, including those induced by oxidative stress caused by high sugar levels (from 14). Information on the role of microRNAs in such cell death, however, is scarce for both the human lens and the lenses of experimental animal models. Therefore, this study represents the first baseline information on their level in the normal mouse lenses and its alteration, induced by feeding a high galactose diet known to induce oxidative stress. Additionally, the effect of supplementing the galactose diet with an antioxidant such as sodium pyruvate on the transcription of miRs has also been determined. It is hypothesized that induction of apoptosis in such lenses may be triggered by the overexpression of pro-apoptotic miRs constituting a stress response to oxidative stress. Table 1 lists the ΔCt values for the miRs that were differentially expressed in the galactosemic and normal lenses, normalized to the housekeeping gene (snRNA), and corrected for the reagent blank and PCR efficiency. It also represents the fold values between the galactose groups and the control group. As may be noted, the individual Ct values in the galactosemic group were substantially higher in 24 miRS and lower in six miRs. On the basis of studies in other systems, all of the upregulated miRs are considered to be pro-apoptotic [29], particularly miR-16, miR-30a, miR-27b, miR-125b, miR-30c, miR-1, and miR-218. In addition, miR 142–3p, miR-15a, miR-29b, miR-22, miR-32, and miR-34c, also known to be pro-apoptotic, are downregulated in the galactosemic lenses as compared to the normals. This downregulation caused by the galactosemic condition may represent a protective response, antagonizing the effectiveness of the highly apoptotic miRs. The response of the tissue in terms of the elevation of miR-21 is intriguing, since this has been reported to be a pro- as well as anti-apoptotic miR [27]. Overall, however, the transcription of pro-apoptotic miRs has been found to be more dominantly expressed.

**Table 1:** List of miRNAs up- or downregulated in galactose stimulated conditions in mice.

**miRNAs**	**Galactose (G1 group) (2^)-∆Ct))**	**Normal (control group) (2^(-∆Ct))**	**Fold regulation**
miR-16	35.63396	4.494815	*7.9278*
miR-21	15.49282	1.787833	*8.6657*
miR-126–3p	2.86875	0.337068	*8.5109*
miR-9	1.892583	0.132899	*14.2408*
miR-30a	15.28937	2.696464	*5.6702*
miR-322	0.818021	0.140242	*5.8329*
miR-27b	6.623755	1.648649	*4.0177*
miR-125b-5p	206.5436	37.87213	*5.4537*
miR-872	3.31476	0.51421	*6.4463*
miR-191	4.507173	1.063679	*4.2373*
miR-126–5p	2.671638	0.155496	*17.1813*
miR-196b	0.000807	0.00005	*16.0325*
miR-150	0.068771	0.014682	*4.684*
miR-10a	0.007942	0.001791	*4.4337*
miR-196a	0.002178	0.0003	*7.2685*
miR-25	3.707246	0.700954	*5.2889*
miR-30c	34.42494	7.572804	*4.5459*
miR-10b	0.001922	0.000213	*9.0104*
miR-411	0.074768	0.013525	*5.528*
miR-295	0.000807	0.000097	*8.3534*
miR-1	109.6921	14.05485	*7.8046*
miR-218	33.58083	4.638208	*7.24*
miR-335–5p	1.255721	0.032472	*38.6711*
miR-374	8.645346	0.845519	*10.2249*
**miR-142–3p**	**0.002516**	**0.015434**	**-6.1343**
**miR-15a**	**0.045813**	**0.203859**	**-4.4498**
**miR-29b**	**0.008401**	**0.037339**	**-4.4448**
**miR-22**	**0.605718**	**4.684158**	**-7.7332**
**miR-32**	**0.028751**	**0.206379**	**-7.1781**
**miR-34c**	**0.067379**	**0.392393**	**-5.8237**

Values in the column Fold Regulation are the normalized gene expression (2^(- ∆Ct)) in the galactose (G1 group) divided by the normalized gene expression (2^(- ∆Ct)) in the normal (control) group. Raw Cts were normalized to housekeeping genes (snRNA), reagent controls, and PCR efficiency. miRNAs that were significantly up regulated by galactose feeding are italicized, and are considered to be primarily apoptotic, except for miR-21, which reportedly can be anti-apoptotic as well. miRNAs in bold are downregulated.

**Figure 1:**
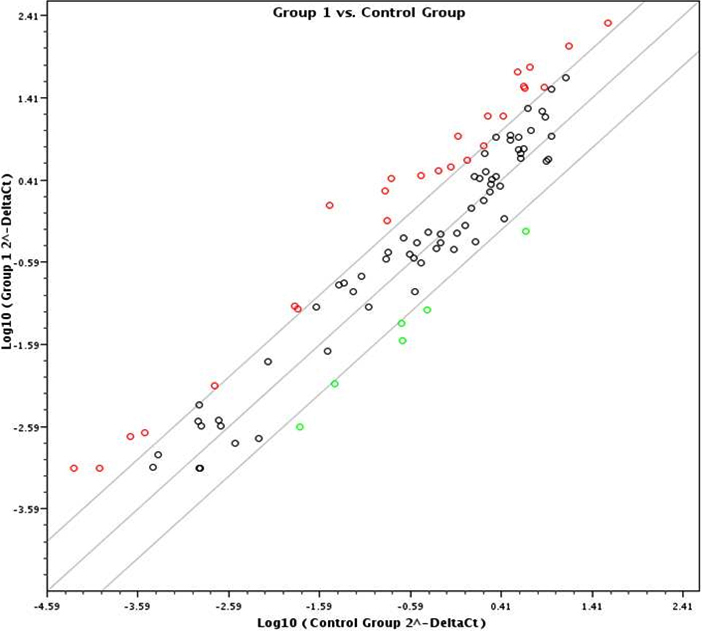
The scatter plot shows the differential expression of miRs between normal and galactose mice lenses. Upregulation is indicated by the red dots and downregulation by the green dots. Galactose-fed (Gr1, y-axis) versus normal (control, x-axis). The plot represents the log_10._2.^(-ΔCt.)^ values of each group.

The significance of the upregulation of the apoptotic miRs is also apparent in the scatter plot (Figure 1) representing the relative expressions of the miRs in the total repertoire of 88 miRs. The individual dots represent the log_10._2^-ΔCt^ values of the miR, the ΔCt of 1 representing a doubling in the number of the cDNA molecules. The majority of the pro-apoptotic miRs are marked with red circles. The repressed ones are represented by green circles. The black circles represent the unaffected miRs. Again, as described in Table 1, there was a significant elevation in the content of pro-apoptotic miRS in the galactosemic lenses with respect to all of the genes present, and not only in those described in Table 1.

With a view toward examining whether the upregulation of the miRs in galactosemic lenses could be accounted for by sugar-induced oxidative stress, a group where the mice were fed a galactose diet containing 1% pyruvate was also simultaneously included. As would be obvious by reference to the relative level of miRs transcriptions in normal versus the galactose plus pyruvate group (Table 2), expression is now significantly repressed as compared to the expression levels in the galactose-alone group summarized in Table 1. Thus, pyruvate is significantly effective in antagonizing the increase in expression of apoptotic miRs caused by galactose. First, fewer genes were found to be expressed. Out of 24 upregulated and six downregulated miRS detected and described in Table 1, there were only 12 upregulated and six downregulated miRNAs. Thus, at least 12 apoptotic genes were significantly downregulated by pyruvate feeding. The repressed genes were also downregulated. As is apparent by the fold regulation values, the upregulation that was found in the case of the 12 miRs was also significantly lower. The repressed genes in the galactose group were also either repressed further or not expressed at all.

**Table 2:** List of miRNAs up- or downregulated in galactose + pyruvate-stimulated condition in mice.

**miRNAs**	**Gal + Pyru (G2 group) (2^(-∆Ct))**	**Normal (control group) (2^(-∆Ct))**	**Fold regulation**
*Mmu-miR-16*	19.73494	4.494815	*4.3906*
*Mmu-miR-21*	9.972084	1.787833	*5.5778*
*Mmu-miR-126–3p*	1.536338	0.337068	*4.5579*
*Mmu-miR-322*	0.723999	0.140242	*5.1625*
*Mmu-miR-872*	2.242205	0.51421	*4.3605*
*Mmu-miR-126–5p*	0.983431	0.155496	*6.3245*
*Mmu-miR-196b*	0.000403	0.00005	*8.0097*
*Mmu-miR-150*	0.061455	0.014682	*4.1857*
*Mmu-miR-196a*	0.001338	0.0003	*4.4645*
*Mmu-miR-1*	68.42511	14.05485	*4.8684*
*Mmu-miR-335–5p*	0.286922	0.032472	*8.836*
*Mmu-miR-374*	3.971576	0.845519	*4.6972*
**Mmu-miR-142–5p**	0.000164	0.0012	**-7.3161**
**Mmu-miR-142–3p**	0.001725	0.015434	**-8.9486**
**Mmu-miR-29b**	0.008598	0.037339	**-4.3427**
**Mmu-miR-32**	0.038068	0.206379	**-5.4213**
**mumiR-144**	0.000096	0.001222	**-12.7342**
**Mmu-miR-214**	0.000418	0.001989	**-4.7579**

The table represents the effect of pyruvate on miRNA transcription in the lenses of galactosemic mice. Numbers in italics represent upregulation of the genes considered to be primarily apoptotic, except for miR-21, which can reportedly act as anti-apoptotic as well as pro-apoptotic. miRNAs in bold are downregulated. Values under the column titled Fold Regulation represent normalized gene expression (2^(- ∆Ct)) in the galactose + pyruvate (G2 group) divided by the normalized gene expression (2^(- ∆Ct)) in the normal (control) group. The raw Cts were normalized to housekeeping genes, reagent controls, and PCR efficiency. It is to be noted that the relative upregulation of the genes in the gal+pyruvate group/normal controls in this case is mostly lower than that caused in the galactose-alone group, as mentioned in Table 1.

**Figure 2:**
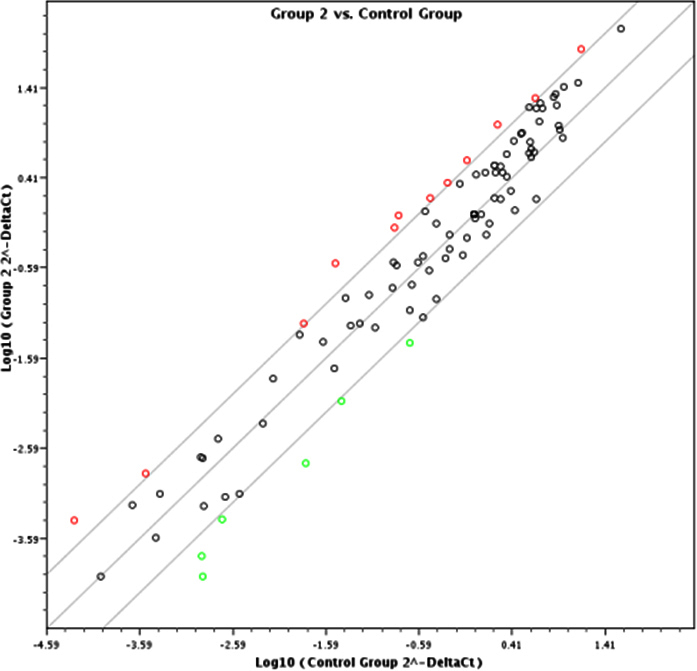
The scatter diagram showing differential expression of miRs between normal and galactose mice lenses. Upregulation is indicated by the red dots and downregulation by the green dots. Galactose+pyruvate fed (Gr2, y-axis) versus normal (control, x-axis).

A global view of the inhibitory effect of pyruvate in limiting the expression of apoptotic miRs induced by galactose is presented in Figure 2. As clearly seen, the number of miRs upregulated in the galactose plus pyruvate group is much lower as compared to those in Figure 1. The numbers upregulated in the galactose plus pyruvate group is only 12, as stated above. However as is also apparent by the position of the red circles, the upregulation is also much lower; the log Ct values remaining closer to the control zone. Most interestingly, the induction of all others was abolished, with six of them becoming even downregulated.

**Figure 3:**
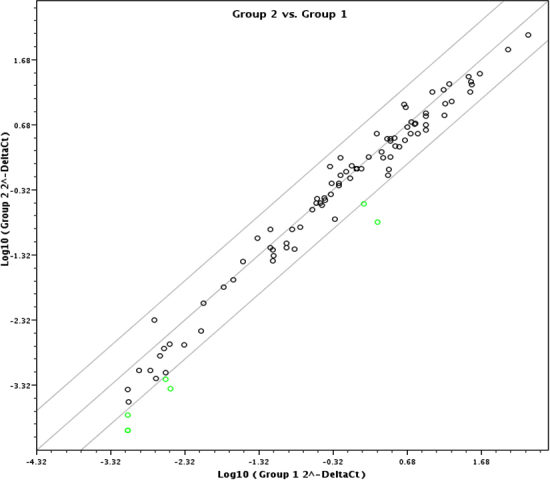
Scatter plot showing the relative expression of miRs in galactose (G1, x-axis) versus gal+pyruvate (G2, y-axis) groups: Downregulation of miRNAs in gal+pyruvate conditions compared to gal alone is indicated by the green dots. The upregulation, if any, was not significant.

A direct comparison of miR expression in the lenses of animals given the galactose diet without and with pyruvate is given in Table 3. In agreement with Table 2, the expression of the apoptotic miRs in the pyruvate + galactose group is, again, significantly lower than in the lenses of mice fed galactose alone. This was apparent also by scatter diagram (Figure 3) comparing the expression levels between the two groups.

**Table 3:** Inhibitory effect of pyruvate on the upregulation of miR transcription induced by galactose feeding.

**miRNAs**	**Galactose (G1 group) (2^(-∆Ct))**	**Galactose+pyruvate (G2 group) (2^(-∆Ct))**	**Fold down regulation**
miR-142–5p	0.000807	0.000164	**−4.9204**
miR-9	1.892583	0.152451	**−12.4144**
miR-144	0.000807	0.000096	**−8.414**
miR-125b-3p	0.002612	0.000586	**−4.4593**
miR-295	0.000807	0.000096	**−8.414**
miR-214	0.003019	0.000418	**−7.2232**
miR-335–5p	1.255721	0.286922	**−4.3765**

The table summarizes the apoptotic genes whose transcriptions were significantly inhibited (downregulated; bold) by adding pyruvate to the galactose diet, the fold regulation being substantially decreased.

## Discussion

It has been previously demonstrated that the apoptotic death of lens epithelial cells is a common feature of most cataracts [44,45]. We have previously demonstrated that it is the case also with sugar cataracts, including that induced by experimental diabetes and galactosemia [13,14,17]. The apoptotic effect has been convincingly demonstrated by electron microscopy showing chromatin degradation and compaction, as well as by the specific molecular DNA changes reflected by enhanced sensitivity to the TUNEL reaction reflecting nick formation, and the pattern of DNA laddering showing oligomers generated by nucleosomal degradation. Treatment of diabetic as well as galactosemic animals with pyruvate has been reported to offset these deleterious effects, along with the overall effect of the prevention of cataract formation. The primary objective of the present studies was therefore to determine whether the apoptotic death associated with cataract formation could be a result of upregulation of certain microRNAs. It is expected that this could induce apoptosis by hybridizing with 3′-UTRs of several mRNAs, consequently limiting their translational activity. In view of the fact that the requirements for hybridization in UTRs is not very stringent in terms of the base complementariness, the increasing number of miRs synthesized representing a stress response to oxidative stress could hybridize non-selectively with a wider range of mRNA species, enforcing the onset of the death pathway even more vigorously. It has been suggested that such miR-induced apoptosis be used therapeutically to kill cancer cells [39,40]. The cells so killed are eventually disposed of by several means, including lymphocytic destruction and proteolysis. In the case of lens, however, no disposal route is available. It is an avascular tissue with no means of removal of the killed cells, and their prompt replacement by new cells is necessary to maintain homeostatic mechanisms.

The killed epithelial cells therefore remain unscavenged and migrate inappropriately deeper into the tissue as well as peripherally under the posterior sub-capsular region. The lack of viable epithelial cells on the lens anterior due to apoptosis also renders the tissue devoid of the bio-energetic support required for nutrient and electrolyte transport, with the consequence of oxidative stress and cataract development. It was therefore considered desirable to study the microRNA expression level of both normal and cataractogenic lenses to examine whether the observed apoptosis associated with cataract development could be explained on the basis of a possible enhancement in miRs transcription in response to oxidative stress. Most importantly, it was decided to examine whether such enhanced transcription could be minimized by certain antioxidants.

These studies have been conducted in mice, as an experimental animal model, given a high galactose diet, known to induce oxidative stress. Such stress is apparent quite early in the history of the disease by the loss of glutathione, which acts as one of its central antioxidant defenses. Thus, additional studies have also been conducted where the animals were fed the galactose diet mixed with sodium pyruvate. This compound is a known scavenger of reactive species of oxygen, and has been previously used as a biocompatible agent in preventing cataract development.

As described in the result section, this is the first study implicating miR transcription in the lens in relation to cataract development. The study was greatly facilitated by the recent availability of PCR cassettes containing primers for cDNA amplification. A 96-well mi-RNA finder cassette containing DNA sequences corresponding to mouse miRs was used. All of the 84 miRs were reproducibly detected (catalogue # MAM-001; SA Bioscience, Frederick, MD).

However, as shown in Table 1, the relative transcription of about 23 miRs was significantly upregulated in the lenses of mice fed the galactosemic diet, as compared to that in the basal controls (normal mice). As shown more clearly in Figure 1, the upregulation was significant to at least 2 log units. Although most of the upregulated genes have been suggested to be pro-apoptotic, a smaller number of genes known to be anti-apoptotic were also noticeably downregulated, perhaps representing a protective response. The protection offered by the latter group apparently falls short of antagonizing the pro-apoptotic effect.

With the help of technological development, the number of miRs expected to be present in vertebrate cells can be as high as 3,000, with highly varied regulatory functions under normal and disease conditions [22,23]. That oxidative stress can induce enhancement as well as downregulation of several miRs associated with consequent cell damage has been found to be true in several pathophysiological situations, such as that caused by ionizing radiation [24], hydrogen peroxide and tert-butyl hydroperoxide [25], liver fibrosis [32], RBC sickling [26], obesity and atherosclerosis [28], etc. Although the regulatory impact of miRs in the development of tissue pathology appears highly pervasive [22,23], dissection of the pathology has been difficult because of the multiple gene targets of the miRs.

The manner in which the reactive species of oxygen may enhance or downregulate miRs also remains unknown. This is partly because of the complexity of factors involved in miRs biogenesis. Canonically, it starts with RNA polymerase II-dependent transcription of the gene, and further processing of the primary gene product by other nucleases, known as Drosha in the nucleus and Dicer in the cytoplasm, to give an initial double-stranded RNA species. Eventually, single-stranded functional miRs are generated in the RISC complex. Information on the effect of antioxidants on such transcription of miRs in vivo, however, is very limited. Our attempt was thus to obtain such information in a physiologic model of oxidative stress in vivo. We selected the model of high sugar-induced oxidative stress. Consequently, further studies were undertaken to examine the possible inhibition in miRs upregulation by pyruvate, which was previously known to scavenge ROS as well as inhibit cataract formation in vivo.

It was therefore interesting to find that the upregulation of the apoptotic miRs in the lenses of galactosemic animals was significantly inhibited by pyruvate given with the galactose diet. As summarized in Table 3, the upregulation of the miRs in galactosemic animals was substantially attenuated in the latter group. The findings also correlate with the decrease in the activities of certain antioxidant enzymes noted in the erythrocytes of humans with cataracts [46] as well as in the lenses [47]. A direct demonstration of the involvement of oxidative stress in the observed miR upregulation has therefore been provided for the first time, at least in vivo. Additionally, these studies may also provide insights for developing anti-miRs suitable for therapy against oxidative stress in lens and other tissues.
